# Non-family Living Arrangements Among Young Adults in the United States

**DOI:** 10.1007/s10680-024-09696-5

**Published:** 2024-03-06

**Authors:** Kristen Jeffers, Albert Esteve, Ewa Batyra

**Affiliations:** 1 Universitat Autònoma de Barcelona, Bellaterra, Spain; 2 Centre d’Estudis Demogràfics, Barcelona, Spain

**Keywords:** Living arrangements, Non-family households, Youth, Gender, Educational attainment, United States

## Abstract

The share of young adults living in married-couple family households in the USA has declined in recent decades. Research on alternative living arrangements focuses on cohabitation among unmarried couples and parent–adult child coresidence. Less is known about trends in non-family living arrangements and the characteristics of young adults living with non-relatives. This study documents trends over time in non-family living arrangements among young adults in the USA and examines the sociodemographic profile of those living with non-relatives. Using pooled US Census and American Community Survey microdata from 1990 to 2019, the authors document age patterns in non-family living arrangements over time and use logistic regression to estimate the likelihood of living with non-family based on individual-level characteristics. Results indicate that non-family living among young adults has increased over time, and that the arrangement is associated with markers of both advantage and disadvantage. Differences across age groups explain some of these mixed results. Trends among younger groups are linked to socioeconomic patterns around family formation. Among older groups, the demographic and labor force characteristics of the foreign born and constraints of their kin availability may be driving trends. The exploratory analysis provides relevant evidence around an increasingly common living arrangement in the USA and also identifies several areas for future research on living arrangements among young adults and the implications of these trends.

## Introduction

Transformations in household composition and living arrangements in the USA have accelerated in recent years. Households are smaller, more people live alone or with unmarried partners, and adult children are prolonging and returning to coresidence with their parents. One of the least studied aspects of these household changes is the rise of non-family living arrangements. According to the 2019 American Community Survey (ACS), nearly 10 percent of individuals aged 20 to 39 live with only non-relatives, yet we know very little about the long-term trends in non-family living arrangements among young adults and the characteristics of individuals living with non-relatives.

To fill this gap, this study undertakes an exploratory analysis of trends in non-family living arrangements among young adults aged 20 to 39 in the USA and the associated individual characteristics. We begin by describing the recent change to patterns of family formation in the USA and how these changes have impacted living arrangements among young adults. We use US Census and American Community Survey (ACS) data collected between 1990 and 2019 to document evolving patterns in non-family family living arrangements over time, identifying persons in non-family living arrangements as those who are not related by kinship or partnership to any other person in the household. We examine the demographic and socioeconomic characteristics associated with non-family living.

Results indicate that non-family living arrangements among young adults aged 20–39 are increasingly common and not only during the ages associated with third-level education. We find that non-family living is associated with markers of both advantage and disadvantage, and that differences across age groups point to parallel profiles of those living with non-relatives that diverge with age. Trends among younger age groups are linked more closely to socioeconomic patterns around family formation. Among older young adults, the demographic and labor force characteristics of the foreign born and constraints of their kin availability drive trends. Our study provides relevant evidence around an increasingly common living arrangement in the USA and also identifies several areas for future research on the diversity of living arrangements among young adults in the USA and the implications of these trends.

## Background

### Shifts in Family Formation Patterns

Shifts in family formation patterns in the USA during the last several decades are well documented in the literature (Cherlin, 2010; Kuperberg, 2014; Lesthaeghe & Neidert, 2006; Manning, 2020; Miller, 2020; Ruggles, 2015; Sassler & Lichter, 2020; Smock & Schwartz, 2020). While trends vary across regions and population groups, overall, the characteristics of family formation in the USA align with the features of the Second Demographic Transition observed also in Western Europe and other high-income countries: delayed marriage and more cohabitation among couples, later and lower fertility, and increased childbearing outside of marriage (Lesthaeghe & Neidert, 2006). The median age at first marriage reached its peak in the USA in 2020, climbing steadily to 30.5 for men and 28.1 for women from 23.2 and 20.8, respectively, in 1970 (U.S. Census Bureau, 2020a). In 1976, fewer than one-third of women aged 25–29 were childless. By 2018, more than half of women this age were childless (U.S. Census Bureau, 2019). Delayed childbearing has contributed to a decline in the average number of children born to women in the USA. While the Total Fertility Rate has been below replacement level (2.1) since 1971, it reached a record low of 1.64 in 2020 (Osterman et al., 2022). Like overall fertility rates, nonmarital fertility rates are declining, but the share of all births to unmarried women has increased from around 10 percent in 1970 to 40 percent in 2020 (Osterman et al., 2022; Ventura & Bachrach, 2000).

These demographic shifts have impacted living arrangements across all age groups but particularly among young adults who represent the age group at which many demographic transitions occur. The transition to adulthood—completing education, beginning full-time employment, leaving the parental home, getting married and having children—occurs later and takes longer now than it did in the period after World War II (Furstenberg, 2010). Delayed and declining marriage and childbearing means that a larger proportion of young adults do not have spouses and/or children with whom to live.

**Fig. 1:**
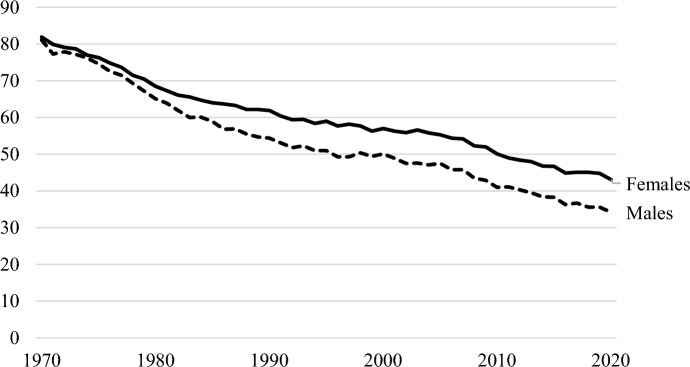
Share of males and females aged 25–34 living with a spouse, with or without children 1970–2020 (%). Source: US Census Bureau, Current Population Survey, Annual Social and Economic Supplement, 1967 to present

Consequently, the share of young adults living in married-couple family households has sharply declined in recent decades (see Fig. 1). In 1970, more than 80 percent of individuals aged 25 to 34 lived with a spouse (with or without a child). By 1995, this had fallen to 55 percent. In 2020, 39 percent of young adults in this age group lived with a spouse (U.S. Census Bureau, 2020b). This trend reflects changes in the household composition across all age groups and provides support for earlier observations of a shortening of the portion of the life cycle during which individuals live with family (Kobrin, 1976) and a growing disconnection between family and households in the USA (Cherlin, 2010). In 1970, more than 80 percent of households in the USA contained families. In 2020, 65 percent of households contained families (U.S. Census Bureau, 2020c).

### Alternatives to Marital Living Arrangements

The alternative living arrangements available to young adults are finite: if one does not live with a spouse or child, one must live with an unmarried partner, with other family members, alone, or with non-family members. Among the alternative living arrangements available to young adults, most research has focused on cohabitation among unmarried couples (Hemez & Manning, 2017; Manning, 2020; Sassler & Lichter, 2020; Sassler & Miller, 2017; Sassler et al., 2018) and, to lesser extent, on the rise in parent–adult child coresidence (Bianchi et al., 2007; Esteve & Reher, 2021; Goldscheider & Goldscheider, 1999; Kahn et al., 2013; Keene 2010; Lei & South, 2016; Mazurik et al., 2020; Sandberg-Thoma et al., 2015). This attention reflects the considerable increase in these living arrangements during the last several decades. In 1975, only one percent of adults aged 25 to 34 lived with an unmarried partner. In 2020, nearly 15 percent of young adults lived with an unmarried partner (U.S. Census Bureau, 2020b). In 2017, 62 percent of women aged 19–44 had ever cohabited compared to one-third in 1987 (Manning, 2020). Parent–adult child coresidence has also increased rapidly, especially in recent years and among males. Between 1980 and 2020, the share of males aged 25 to 34 living with a parent nearly doubled from 11 to 21 percent (U.S. Census Bureau, 2020b). This is now the most common living arrangement among unmarried young adult males. Living alone has also become more common. The share of young adults living alone has more than doubled since 1970 (U.S. Census Bureau, 2020b). In 2020, 12 percent of men and 9 percent of women aged 25 to 34 lived alone (U.S Census Bureau, 2020b).

Changes in living arrangements and family formation have been linked to attitudinal and economic changes. Cohabitation among unmarried adults has become more accepted in American society, and cohabitation more often than marriage is now a primary marker of emerging adulthood for young people (Furstenberg, 2015; Manning & Cohen, 2015; Sassler & Litcher, 2020). The timing and sequence of the transition to adulthood differ by socioeconomic status (Furstenberg, 2008). Socioeconomic differences are also driving trends in cohabitation. Women from advantaged backgrounds are more likely to marry and less likely to cohabit, and college-educated women are more likely to transition from cohabitation to marriage than less-educated women (Manning et al., 2014; Sassler et al., 2018; Smock & Schwartz, 2020). Economic stability has become a prerequisite to marriage. Many young people expect to reach a certain level of job security and income before they marry (Furstenberg, 2015; Ruggles, 2015). In the last 40 years, real wages among young men and women have declined in the USA (Ruggles, 2015). These economic barriers contribute to delayed marriage and increased cohabitation and provide support for the diverging destinies framework which highlights differences in demographic change across socioeconomic groups (McLanahan, 2004; Smock & Schwartz, 2020).

Economic conditions are also driving the increase in parent–child coresidence in the last several decades, a trend that has accelerated rapidly since the Great Recession. The share of young men aged 25 to 34 living with a parent increased from 15 to 21 percent between 2010 and 2020, and the economic downturn associated with the Covid-19 pandemic has likely contributed to further increases since 2021. Challenging the historical understanding of intergenerational coresidence, the arrangement has become associated with economic need among the younger not the older generation (Kahn et al., 2013; Matsudaira, 2016; Ruggles, 2007). In 1960, adult children with more resources were more likely to live with parents. In 2010, adult children with fewer resources were more likely to live with parents (Kahn et al., 2013). Declining wages, rising housing costs, and student debt have contributed to a deteriorating economic position for young people in the USA which has driven them back to their parental homes (Houle & Warner, 2017; Matsudaira, 2016; Ruggles, 2015). As is the case with cohabitation among unmarried partners, there are important socioeconomic differences. Young adults who are non-white or have low education are more likely to live with a parent (Kahn et al., 2013). This mirrors population-level trends—intergenerational and extended-family households are more common among non-white and low-SES individuals of all ages in the USA (Amorim et al., 2017; Cross, 2018; Harvey & Dunifon, 2023; Kamo, 2000; Pilkauskas, 2012; Pilkauskas & Cross, 2018; Pilkauskas et al., 2020; Swartz, 2009; Reyes et al., 2020).

### Non-Family-Based Living Arrangements

There is comparatively less research on another increasingly common living arrangement among young adults—non-family living. In 2019, nearly 10 percent of individuals aged 18 to 24 and 7 percent of individuals aged 25 to 34 lived with non-relatives only (U.S. Census Bureau, 2020b, 2020e). The limited literature that has explored trends in non-family living in the USA has (Christian, 1989), by necessity, included cohabiting unmarried partners in this category of living arrangement: before 1990, the US Census did not distinguish unmarried partners from other non-relatives in relationship data. Now that it is possible with available data, it is important to examine coresidence with non-relatives separately from cohabitation.

The existing evidence tells us little about how cultural and economic factors have influenced the rise in non-family living arrangements. One may think that in an individualistic society like the USA, living independently—that is, alone or with a spouse or partner—is preferred to living in an intergenerational or extended-family household, living arrangements that are generally associated with economic disadvantage. Where exactly non-family living arrangements fall on the spectrums of independence and affluence is not clear. Among older adults, non-family living may be a last resort for the economically disadvantaged. However, young adults living with non-relatives could represent a relatively privileged segment of the population. Well-educated young adults are more likely to be unmarried and childless, giving them the option to live with non-relatives (Furstenberg, 2008). Living away from the parental home before marriage and/or childbearing is an increasingly important rite of passage (Rosenfeld, 2007), and living with non-relatives might represent a lifestyle choice among young adults which provides social as well as financial benefits (Heath & Kenyon, 2001; Stone et al., 2011). Some recent descriptive evidence on non-family households is available from the Census Bureau, but we know little about the individual characteristics associated with the arrangement.

One way to understand whether non-family living is a response to economic need or reflects a preference regardless of socioeconomic status is to examine the profiles of individuals in this arrangement. Among the several factors that might be driving the trends in non-family living arrangements among young adults, three stand out as having potentially strong and direct effects: education, migration, and income.

The share of the population pursuing university and post-graduate study has increased in the USA with the potential to impact living arrangements in several ways. Many college students live in university collective housing or share private dwellings with classmates. Students are also likely to delay family formation until they have completed their studies. An increase in the number and age of students could therefore prolong periods of university-related non-family living arrangements. The university educated are also more likely to work in specialized professions that require relocation and to pursue professions concentrated in large urban areas with high rents. Indeed, in their study of living arrangements among young adults in the UK, Stone and colleagues (2011) find that living with non-relatives is more common among students or individuals with a previous experience of higher education. We therefore take into account the influence of educational expansion by restricting our analysis to non-family living arrangements in private households (excluding group quarters such as university dormitories) and including measures of educational attainment and school attendance in our analysis.

Migrants—both international and internal—have historically been more likely to live in complex and non-family households (Van Hook et al., 2007). The availability of kin is restricted for migrants for a number of reasons: young migrants are more likely to be single and childless and migrants of all ages are more likely to live apart from partners, children, and other family members. Furthermore, migrants are more likely to work in low-wage jobs that do not afford independent living, especially in large urban areas with high housing costs. Stone and colleagues (2011) find that the increase in non-family living among young men in the UK is largely explained by immigration-driven changes in the composition of the population.

The need to share housing with non-family members is not unique to migrants. In many cities in the USA, housing costs prohibit independent living. Journalistic evidence suggests it is becoming increasingly common for cohabiting or married couples—even those with children—to live with roommates to help defray high rents (Herron, 2019; Moylan, 2019; Pappu, 2016). Our analysis explores living arrangements such as these from the perspective of the roommate. In line with the literature on other forms of non-independent living arrangements, we expect non-family living to be less common among those with higher incomes. We include measures of migration, income, and urban residence in our analysis.

To complement the literature on other living arrangements among young adults, we address several original questions: What share of individuals live in non-family arrangements across age groups and has this changed over time? Are trends driven by educational expansion and migration? Do non-family living arrangements reflect preferences or necessity? Are they associated with affluence or disadvantage? To answer these questions, we examine trends in the share of young adults living with non-relatives over time and determine which individual characteristics are associated with living in non-family households. Given our interest in individual demographic transitions, we focus our investigation on the period of early adulthood between the ages of 25 and 29 during which most individuals begin to establish independent households, but we present results for other age groups as well.

## Data and Methods

Our interest is in shared living arrangements among individuals without family or kin-like ties. We consider non-married partners to have a kin-like relationship and exclude them from our definition of non-family living. The unitary model of household utility proposed by Gary Becker (1981) has been challenged for family households, and this model is even less applicable to households of non-relatives where the pooling of resources and uniform preferences are less likely. For this reason, we study individuals rather than entire households. We identify individuals living in non-family arrangements as those persons living in multi-person private households who are not related to anyone in the household, without distinguishing whether the other members are related or not.

This study pools US Census microdata for 1990, 2000, and American Community Survey (ACS) microdata for the years 2010 and 2019. These data provide the longest available view of change in the prevalence of non-family living in the USA. The 1990 census is the earliest to distinguish non-married partners from other non-relatives, and the 2019 ACS is the most recent national survey that is sufficiently comparable to earlier data. Microdata for the 2020 ACS were available at the time of writing, but due to the effects of the COVID-19 pandemic on data collection and data quality, the Census Bureau released the 2020 1-year ACS PUMS file with experimental weights and cautioned that the data are not comparable to other ACS sample years. Furthermore, the COVID-19 pandemic disrupted the living situations of many individuals—especially college students and young adults. Data from 2020 are therefore not comparable to previous years or useful for studying long-term trends. These sample years provide roughly equally spaced observations across four decades. The microdata samples were accessed through IPUMS and provide self-weighted, nationally representative 5 percent samples of the 1990 and 2000 censuses, and 1 percent samples of the ACS (Ruggles et al., 2021, https://usa.ipums.org/usa/).

In these microdata samples, individuals are organized into households, allowing for the analysis of household structure and classification of household type. To identify individuals in private non-family living arrangements, we rely on the relationship to household head variable, RELATE, and the IPUMS-constructed family interrelationship variables that identify each individual’s co-resident spouse (opposite or same-sex), mother, and father. The RELATE variable indicates if and how each individual living in the household is related to the household head. The interrelationship variables allow us to identify family ties among individuals not related to the head.

IPUMS assigns links between family members in the family interrelationship variables based on the information reported in the RELATE variable as well as age, sex, marital status, and the order in which individuals are listed on the census and survey forms. (More information on the IPUMS family interrelationship variables is available from the IPUMS website and in Ruggles 1995.) To illustrate the value of these family interrelationship variables, take the household presented in Table 1 as an example. The empirical relationship information collected in the census or ACS is in reference to the household head, and the relationships among persons 2, 3, and 4 are not documented in any variable reflecting data collected directly from respondents in questionnaires. Nonetheless, the age, sex, and marital status information of these individuals as well as their proximate order in the household roster point to family ties. The IPUMS samples use this information to link these individuals and generate “pointer” variables indicating the location in the household of spouse and parents. Using these variables, we can identify more family relationships than based on relationship to head alone.

**Table 1:** Family relationships in IPUMS

Person	Relationship	Age	Sex	Marital status	Spouse	Mother	Father
1	Head	64	F	Widow	0	0	0
2	Non-relative	31	M	Married	3	0	0
3	Non-relative	29	F	Married	2	0	0
4	Non-relative	4	F	Single	0	3	2

We use a process of elimination to identify individuals living with non-relatives. We first exclude all individuals who are related to the head of the household. We make no distinctions between primary kin and extended family. Any individual related to the head by blood or marriage is considered to live with family. The heads of these households are also excluded. Foster children are distinguishable from other non-relatives beginning in 2000. In our analysis, foster children are considered non-relatives, but they represent a very small percentage of individuals in the age groups of focus. Next, we exclude individuals for whom co-resident spouses, mothers, fathers, or children have been identified based on the interrelationship pointer variables. The individuals that remain fall into two categories (1) individuals who are not related to the head of household and no spouse, mother, father, or child has been identified in the household and (2) heads of households that contain only non-relatives of the head (person 1 in Table 1, for example). We classify these individuals as living in non-family arrangements. The pooled sample consists of 32,002,596 individuals and 12,348,163 households. Individuals living in households where no one is related—“roommate” households—represent 71 percent of the sample, and individuals not related to anyone in a household with related persons—persons “hosted” by a family household—represent 29 percent of the sample. Results presented in Sect. 4 are consistent whether or not individuals in the second group are included.

We do not capture every family relationship with this approach. We do not identify persons unrelated to the head but related to one another in ways other than parent–child or spouse. For example, if two siblings or cousins live together in a household with other individuals and neither of the pair of siblings or cousins is related to the household head, it is not possible to identify this family relationship. These individuals are not excluded in our process of elimination and are counted among those living in non-family arrangements. This could lead to a slight overestimation of the size of the population living with non-relatives only.

The analysis consists of two parts. The first phase documents trends in non-family living arrangements in the USA by age group across time. Anticipating both age and period effects, we document age patterns in non-family family living arrangements and how these patterns have changed across time. The second phase of the analysis relies on logistic regression to estimate the likelihood of living with non-family when we control for individual-level demographic and socioeconomic characteristics.

We focus our analysis on the period of early adulthood between the ages of 25 and 29 during which most individuals begin to establish independent households. Results are also presented for the 20–24, 30–34, and 35–39 age groups.

We run two logistic regression models each for males and females to examine changes over time with and without adjustments for other sociodemographic characteristics. The dependent variable in the models is an indicator of whether the individual lives with non-family only. Model 1 considers period effects only with census or survey year as the only independent variable. In Model 2, in addition to census or survey year, we include other individual characteristics as potential correlates of an individual’s likelihood of being in a non-family living arrangement. Marital status includes three categories: (1) never married, (2) married, and (3) separated, divorced, or widowed. We combine IPUMS race and Hispanic origin variables into a single measure with five categories: (1) non-Hispanic White, (2) non-Hispanic Black, (3) non-Hispanic Asian, (4) non-Hispanic other race, and (5) Hispanic/Latino of any race. Nativity is dichotomized based on the individual’s place of birth: (1) native (born in the USA, excluding outlying areas and territories) and (2) foreign-born. School attendance corresponds to the IPUMS variable which indicates for individuals aged three years and older if he or she was attending nursery school, kindergarten, elementary school, or any schooling leading toward a high school diploma or college degree. Educational attainment categorizes individuals based on highest year of school or degree *completed* at the time of the survey or census: (1) primary or less: grade 11 or below completed, (2) secondary: grade 12 completed or up to three years of college completed, (3) four years or more of college completed. Employment status indicates whether the individual was (1) employed, (2) unemployed, or (3) not in the labor force during the week before the census or ACS was completed. We categorize whether the individual’s household was located in a metropolitan area according to the IPUMS metropolitan status variable, which uses the US Census Bureau definition: a region consisting of a large urban core together with surrounding communities that have a high degree of economic and social integration with the urban core. Poverty status indicates if the individual had a family total income for the previous year (1) below the official poverty threshold, (2) between 100 and 199 percent of the poverty threshold, or (3) 200 percent of poverty threshold or above. Poverty status is determined at the family—not the household—level. Therefore, for adults living as unrelated individuals, poverty status reflects individual income.

We have not included interaction terms in our model. We explored interactions between independent variables and did not observe strong categorical differences in the effect of independent variables on one another.

The variables included in the analysis are highly comparable across the samples pooled for analysis. Basic demographic variables such as age, sex, and marital status are completely comparable for our purposes. Practices around the collection of race and educational attainment information have been generally consistent across censuses and ACS in the USA since 1990. IPUMS-constructed income and poverty variables provide cross-temporally comparable measures that account for inflation and changes to poverty thresholds. Likewise, the IPUMS-constructed variable that identifies households in metropolitan areas applies consistent metropolitan area definitions across all samples included in the analysis.

## Results

We present results for the analysis of non-family living arrangements in the USA by age group and across time separately for males and females. Table 2 reports the percent living with non-family for men and women at ages 20–24, 25–29, 30–34, and 35–39 by census or survey year. The share of individuals living with non-family has increased over time at every age. Change over time has been monotonic at most but not all ages. The share of individuals in non-family living arrangements is highest in 2019 for all age groups except for men aged 20–24. Among men aged 20–24, the share living with non-family is highest in 2010. Nonetheless, the share of men aged 20 to 24 living with non-family is higher in 2019 than in 1990. We do not observe this pattern among men at other ages or among women, for whom there has been a steady increase between the earliest observation and the most recent observation at all ages. Men are more likely than women to live with non-family at all ages, and the difference in the share of living with non-family between the earliest and most recent observations is greater for men than for women across the 25–29, 30–34, and 35–39 age groups. The difference between the earliest and most recent observations is greater for women than men at age 20–24. The largest increases over time are observed at ages 25–29 for men and ages 20–24 for women. Between 1990 and 2019, the share of men living with non-family at ages 25–29 increased by 4.3 percentage points. For women aged 20–24, the share living with non-family increased by 4.2 percentage points between 1990 and 2019. The change over time is less pronounced at ages 30–34 and 35–39 and remains slightly larger for men than for women.

**Table 2:** Percentage of males and females aged 20 to 39 living in non-family households by 5-year age group and census or survey year

Year	Males				Females			
	20–24	25–29	30–34	35–39	20–24	25–29	30–34	35–39
1990	15	10	5.6	3.9	12.1	6.3	3.4	2.4
2000	18.3	12.2	7	4.6	14	7	3.6	2.7
2010	18.4	13.7	8	5.6	15.6	8.4	4.2	2.8
2019	17	14.3	9	5.7	16.3	10.1	5	3.4

Table 3 provides sample characteristics and presents the share of men and women in non-family living arrangements by covariates included in the logistic regression models and census or survey year for the 25–29 age group. (Sample characteristics and bivariate relationships for other age groups are available in Appendix Tables 5 and 6.) Considerable changes in the composition of the sample population over time are observed. The share of 25-to-29-year-olds completing university has increased, with a larger increase for females. School attendance increased between 1990 and 2010 and decreased slightly between 2010 and 2019. Employment has decreased among men but increased among women. The relative size of the foreign-born population increased significantly between 1990 and 2010 and has decreased since. The share of the population that is white has decreased steadily. Among the covariates considered, the largest compositional change has been around the share of individuals who are single at ages 25–29. Nearly three-quarters of men and two-thirds of women were single at this age in 2019, compared to less than half of men and less than one-third of women in 1990. The share of men and women living in urban areas has increased, but only slightly. Poverty was highest among both men and women in 2010 and has decreased since. Across all years, women are more likely than men to experience poverty at this age.

**Table 3:** Sample characteristics and share in non-family households by covariate and census or survey year, males and females aged 25–29

	Males		Females	
	% of total sample	% living in non-family household	% of total sample	% living in non-family household
*University complete*				
1990	22.3	15.1	22.5	11.1
2000	25.6	16.4	29.9	10.9
2010	27.1	18.4	35	12.5
2019	32.5	19.9	39.9	13.9
*Attending school*				
1990	13.7	9.1	13.6	5.7
2000	13.6	11.4	15.1	6.3
2010	15.4	13.1	19.5	7.9
2019	12.7	13.6	16.1	9.5
Employed				
1990	87	9.9	69.9	7.5
2000	83.2	12.1	69.7	7.8
2010	78.6	14.3	69.7	9.5
2019	84.7	14.8	77.3	11
*Foreign-born*				
1990	12.9	14.9	11.4	7.9
2000	20.7	16.6	18.5	8.2
2010	19.8	19.5	18.7	8.9
2019	15.2	19.1	15.3	11.3
*Non-Hispanic white*				
1990	74	9.8	72.8	6.5
2000	63	11.6	62.4	7.4
2005	59.6	11.5	60.2	6.9
2010	59.6	13.4	58.1	9.2
2015	57.4	16	56.3	11.5
2019	55.2	14.8	54	11.1
*Single*				
1990	44.2	19	31.2	16
2000	49.7	20.7	38.2	15.1
2010	63.8	19.4	51.8	14.3
2019	71.8	18.6	61.5	15.1
*Living in metropolitan area*				
1990	79.6	11.6	79.3	7.3
2000	80	13.4	80.1	7.8
2010	81	14.8	81.1	9.3
2019	83.6	15.3	83.6	11
*Income below poverty threshold*				
1990	10	17.9	14.2	9.7
2000	9.9	22.4	14.6	11.2
2010	13.5	23.2	19.7	12.1
2019	8.9	26.8	14.6	16.8
*Total*				
1990		10		6.3
2000		12.2		7
2010		13.7		8.4
2019		14.3		10.1

The bivariate results presented in Table 3 tell a somewhat paradoxical story about the relationship between socioeconomic status (SES) and the likelihood of living with non-family only. Higher absolute levels in the proportion of people in non-family living arrangements and larger changes over time are observed for specific subgroups, but trends across characteristics are not consistent with typical conceptualizations of advantage and disadvantage. Compared to the total population ages 25–29 living with non-family (presented at the bottom of Table 3), men and women with a university degree are more likely to live with non-family, implying the living arrangement may be associated with higher SES. Among men, the change over time for those with a university degree is larger than for the total population in this age group. The change over time is about the same among university-educated women and the total population. Non-family living arrangements are associated with university students, but across all years individuals attending school are less likely to live with non-family at this age (females only slightly so) compared to the total population.

The period gradient among those who were employed, were non-Hispanic white, or were living in urban areas largely mirrors trends for the total population, though the share of individuals living in non-family arrangements in these groups was slightly higher compared to the overall population in all years. The share living with non-family is higher for foreign-born individuals than the total population in all years. In 2010 and 2019, nearly 20 percent of foreign-born men lived with non-relatives only. Incongruous with trends around educational attainment, non-family living is much more common among individuals with incomes below the poverty threshold. More than one quarter of men (27 percent) and 17 percent of women with incomes below the poverty threshold lived in non-family households in 2019. The difference over time is much more pronounced for individuals with incomes below the poverty level than for the total population aged 25–29 living with non-family as well, with the share increasing by nine and seven percentage points between 1990 and 2019 for men and women, respectively. The share of single people living in non-family arrangements is higher than for the total population, but no pattern across time is discernible.

Table 4 presents the odds ratio results of logistic regression models predicting the likelihood of non-family living for men and women aged 25 to 29. Model 1 includes census or survey year as the only independent variable and confirms higher odds of non-family living in recent years compared to earlier years for both men and women. The odds of living with non-family are 1.5 times higher among males in 2019—compared to the earliest observation in 1990. Among females, the odds of living with non-family are 1.7 times higher in 2019 compared to 1990.

**Table 4:** Results of logistic regression of likelihood to live in a non-family household for males and females aged 25–29

	Odds ratio (SE)			
	Males		Females	
Variable	Model 1	Model 2	Model 1	Model 2
Year				
1990 (reference)	1	1	1	1
2000	1.243 (0.002)	1.158 (.002)	1.117 (.002)	0.924 (.002)
2010	1.427 (0.002)	1.049 (0.002)	1.372 (0.002)	0.847 (0.002)
2019	1.495 (0.002)	1.047 (0.002)	1.687 (0.003)	0.934 (0.002)
Educational attainment				
Primary or less (reference)		1		1
Secondary		1.095 (0.002)		1.109 (0.003)
University		1.890 (0.003)		2.198 (0.006)
School attendance		1.164 (0.001)		1.126 (0.002)
Employment status				
Employed (reference)		1		1
Unemployed		0.493 (0.001)		0.598 (0.002)
Inactive		0.538 (0.001)		0.632 (0.001)
Foreign-born		1.755 (0.002)		1.509(0.003)
Race and ethnicity				
White (reference)		1		1
Non-Hispanic Black		0.561 (0.001)		0.364 (0.001)
Non-Hispanic Asian		0.861 (0.002)		0.964 (0.003)
Non-Hispanic other		0.779 (0.002)		0.765 (0.003)
Hispanic any race		0.764 (0.001)		0.629 (0.001)
Marital status				
Single (reference)		1		1
Married		0.064 (0.000)		0.046 (0.000)
Divorced, separated, widowed		0.869 (0.001)		0.583 (0.001)
Residence in metropolitan area		1.647 (0.002)		1.803 (0.004)
Poverty status				
Below threshold (reference)		1		1
Income 100–200% of threshold		0.578 (0.001)		0.655 (0.001)
Income > 200% of threshold		0.296 (0.000)		0.383 (0.001)

**p < 0.001 for all variables**

When we control for individual-level demographic and socioeconomic characteristics in Model 2, the increases over time disappear, suggesting that the increase in non-family living arrangements among young adults is largely explained by changes in the composition and characteristics of the population over time. Consistent with the bivariate analysis, level of education is strongly positively associated with non-family living. The odds of living with non-family are nearly twice as high for men who have completed four or more years of college compared to those who have completed less than secondary education. Among women, those with a university degree have odds of living in a non-family arrangement that are 2.2 times higher than for their less-educated peers. Attending school increases slightly the odds of non-family living for both men and women. Working men and women have higher odds of living in non-family arrangements than the unemployed or those outside the labor force. The odds of living with non-family are 50 percent higher among employed men than among unemployed men. Among women, the odds of living with non-family are 40 percent higher among the employed compared to the unemployed.

Non-family living arrangements are more common among those born outside the USA than the native-born. The odds of living with non-family only are 1.8 times higher for foreign-born men than for native-born men, and the odds for foreign-born women are 1.5 times higher than for native-born women. There are significant differences by race. The odds of non-family living are higher among white individuals than among other racial and ethnic groups. The odds of non-family living are lowest among the black and the Hispanic populations. The odds of living with non-family are 60 percent as high among non-Hispanic black men and 80 percent as high among Hispanic men compared to white men. The odds are 40 percent as high for non-Hispanic black women and 60 percent as high for Hispanic women compared to white women. Not surprisingly, the odds of non-family living are vastly lower among married individuals than among individuals who have never been married. Divorced, separated, or widowed men have odds of living with non-family only that are 10 percent lower than never married men. The odds among previously married women are 40 percent lower than among never married women.

Non-family living is an urban phenomenon. Living in a metropolitan area significantly increases the odds of non-family living for both men (odds ratio = 1.65) and women (odds ratio = 1.80). Income is strongly and negatively associated with non-family living for both men and women. Those with personal income levels below the poverty threshold have odds of living with non-family that are 60 (females) to 70 (males) percent higher than those with incomes that are more than twice the poverty threshold.

We also ran Models 1 and 2 for the 20–24, 30–34, and 35–39 age groups (see Appendix Tables 7, 8, 9). The patterns observed for the 25–29 age group are largely mirrored in the other age groups with some notable exceptions. Among men and women aged 20 to 24, education and income levels are the strongest predictors of living with non-family, and the relative effect of nativity status is lower compared to other control variables. Among the older age groups, the effect of education is smaller relative to other control variables. Marital status, nativity status, and income level are the strongest predictors of non-family living among those aged 30 to 34 and 35 to 39. For these age groups, level of education has less importance relative to most other control variables. At ages 35–39, education is negatively associated with non-family living for men. The odds of living with non-family are slightly higher among non-Hispanic Asian women than among white women at ages 30–34 and 35–39.

## Discussion

This study has investigated trends in non-family living arrangements among young adults in the USA and sheds light on the individual characteristics associated with non-family living. Despite the availability of data to support such an investigation, ours is the first study to examine changes over time in non-family living arrangements as well as the determinants of living with non-relatives. Our analysis was largely exploratory, and our results have both provided important empirical evidence around an increasingly common living arrangement in the USA and generated new questions for future research.

We find that non-family living is becoming more common in the USA. The share of individuals living with non-relatives only has increased over time at every age studied. Relative age trends have remained consistent, with the largest share of men and women in non-family living during the ages most associated with university attendance and then decreasing with age. Over time, the post-university decline in the share of young adults living in with non-family has flattened for both men and women but especially for men. The largest increase over time has been for the 25 to 29 age group. Consistent with studies of other living arrangements among young adults, this finding suggests more recent birth cohorts are delaying the formation of independent households.

The increase in non-family living arrangements among young adults is explained by changes in the composition and characteristics of the population over time. University education is the strongest predictor of non-family living for men and women ages 20 to 25 and 25 to 29. Viewed in the context of rapid educational expansion across the years captured in our sample, this finding might suggest that the increase in non-family living simply reflects the prolonging of third-level education as more young adults pursue postgraduate study and remain in student living arrangements. Indeed, school attendance is associated with non-family living, with a strong effect among 20- to 24-year-olds.

A more complex story emerges when we consider the other characteristics examined. Employment status is a stronger predictor of non-family living than school attendance at all ages for men and women. Living in a metropolitan area, being White, being foreign-born, and having a personal income below the poverty threshold are highly associated with non-family living for all age groups. These results indicate that non-family living is associated with markers of both advantage and disadvantage. On the one hand, having a four-year college degree, being employed, being White, and living in an urban area are characteristics associated with relative socioeconomic advantage in the USA. At the same time, the strong effects of poverty status suggest that the living arrangement is one of necessity rather than choice. Differences across age groups in the relative effect of control variables explain some of these mixed results and point to parallel profiles of individuals living with non-family that diverge with age. Our results suggest that non-family living is driven by economic necessity in urban areas among those not living with spouses, partners or children. This population is made up of single, childless people and people living apart from their families, and results are in line with our understanding of social and economic stratification of family formation throughout the life course.

At younger ages (20 to 24), the characteristics of those most likely to be living with non-family are in line with the characteristics of people we expect to be single and childless: college-educated Whites. The strong effect of being college educated relative to other control variables and the large differences across racial and ethnic groups are consistent with what we know about socioeconomic trends in family formation and living arrangements. Highly educated people cohabit, marry, and have children later than the less educated. White and Asian individuals are more likely to have a 4-year college degree than other racial and ethnic groups. Non-white individuals are more likely to live in multigenerational or complex family households and tend to marry/cohabit (Hispanic) and/or have children earlier (Black). These results point to one profile of individuals living in non-family arrangements: college-educated young white adults residing in urban areas earning relatively low wages as they begin careers. The poverty status variable used in our analysis does not capture intergenerational transfers of wealth or financial support received from other family members, which may be a significant source of finances among younger adults. The young adults living with non-family may have more financial resources available to them than what we are capturing in our models.

The foreign-born represent a parallel profile of those living with non-family. At all ages, the foreign-born are more likely than the native-born to live with non-family. At younger ages, the foreign-born may more closely resemble the overall population when it comes to the characteristics included in our model and their relationship with family formation, explaining the weaker effect of nativity relative to other characteristics like education level, school attendance, and income level for those aged 20 to 24. Among men aged 35 to 39, nativity is the strongest determinant of non-family living after marital status. At the same time, the relative effect of education, poverty status, and race change with age. This may reflect less pronounced social and economic stratification of family formation among older individuals. Most individuals are partnered and have children by the mid-30 s, and gaps by educational level and race diminish greatly. These results suggest that at older ages, the foreign-born may be the least likely group to live with a partner, child, or other family members due to later marriage among foreign-born (Mayol-García et al., 2021) and the higher likelihood that the foreign-born live apart from spouses and children for periods of time. This interpretation is further supported by the fact that among those age 30 to 34 and 35 to 39, Hispanic men and Asian women are nearly as or more likely than white men and women to live with non-family only. The limitation of our analysis to capture all intrahousehold family relationships may be a contributing factor here as well. Previous studies (Van Hook et al., 2007) suggest that the foreign-born are more likely to live in complex households that include more distant family members—relationships we do not identify in all cases.

Educational expansion, delayed marriage and cohabitation, increased immigration and urbanization have increased the share of the population with the characteristics associated with non-family living. Trends among younger age groups are linked more closely to socioeconomic patterns around family formation. Among older age groups, the demographic and labor force characteristics of the foreign born and constraints of their kin availability may be driving trends.

Our findings highlight some of the complexity of non-family living arrangements and generate a multitude of questions on the diversity of living arrangements among young adults in the USA and the implications of these trends. As this paper explores non-family living in isolation of other living arrangements, future investigations could consider non-family living alongside cohabitation with spouse or partner, parent–adult child coresidence, living alone, and residence in collective housing to provide a better understanding of how young adults across these living arrangements differ from one another. Individuals living in households where no one is related—“roommate” households—represent the majority of our sample, and results of our exploratory analysis are consistent whether or not individuals “hosted” by a family are also included. Future research could examine differences between these two groups of non-family dwellers. Studies could further explore aspects of these different types of non-family living arrangements: in “roommate” only households, do all household members have similar demographic and socioeconomic characteristics? When an individual lives with a family that he or she is not related to, what are the relative situations of the “host” family and the non-family “guest” (like the work done by Harvey & Dunifon, 2023 on households where parents and their children live with extended family). Another approach could be to compare results across a typology of complex households that contain at least one non-family member. Finally, foreign-born refers to an increasingly diverse population in the USA. Further study focused on this population is required to better determine what drives non-family living across migrant characteristics.

## Data and Material Availability

The datasets generated during and analyzed during the current study are available in the IPUMS USA database: https://usa.ipums.org/usa/.

## Appendix

See Tables

**Table 5:** Sample characteristics and share in non-family households by age group, covariate, and census or survey year, males

	% of total sample				% living in non-family household			
	20–24	25–29	30–34	35–39	20–24	25–29	30–34	35–39
University complete								
1990	10	22.3	24.6	28.5	23.6	15.1	6.1	3.5
2000	9.6	25.6	27.7	26.3	27	16.4	7.1	3.7
2010	11.2	27.1	29.5	30.2	25.7	18.4	7.6	3.6
2019	14.6	32.5	35.5	35.3	23.5	19.9	9.4	4.1
Attending school								
1990	30.9	13.7	8.8	6.7	7.9	9.1	5.4	3.9
2000	30.8	13.6	8.0	5.6	9.8	11.4	6.8	4.7
2010	37.3	15.4	8.6	5.6	10	13.1	7.9	5.5
2019	35	12.7	7.1	4.6	9.7	13.6	8.8	5.6
Employed								
1990	75.7	87	89.2	89.7	10.8	9.9	5.4	3.7
2000	73.5	83.2	85.3	85.5	12.4	12.1	6.7	4.4
2010	64	78.6	82.5	84	13.7	14.3	7.8	5.1
2019	72.5	84.7	87.9	88.4	13.3	14.8	8.9	5.4
Foreign-born								
1990	12.5	12.9	12.3	11.5	19.4	14.9	9.2	6.6
2000	17.9	20.7	20.2	17.8	20.7	16.6	10.4	7.4
2010	15.4	19.8	23.3	24.3	20.7	19.5	12	8.5
2019	11.8	15.2	20.2	23.2	21.3	19.1	11	7.8
Non-Hispanic white								
1990	71.4	74	76.2	77.9	15.8	9.8	5.1	3.5
2000	61.3	63	66.1	69.9	20.1	11.6	6	3.9
2010	57.3	59.6	59	60.4	21.2	13.4	6.8	4.3
2019	53.9	55.2	56.4	57.2	19.8	14.8	8.5	4.8
Single								
1990	77.3	44.2	24.3	14.7	18.4	19	15.4	13.6
2000	80.3	49.7	29.6	20.1	21.4	20.7	16.6	13.5
2010	89.5	63.8	38.5	25.3	20	19.4	15.4	13.2
2019	91.7	71.8	47.5	30.9	18.1	18.6	15.9	12.5
Living in metropolitan area								
1990	79.8	79.6	76.9	74.4	17.4	11.6	6.6	4.8
2000	78	80	80	78.5	18.9	13.4	7.8	5.3
2010	79.2	81	80.3	79.4	19	14.8	8.7	6.1
2019	81.5	83.6	84.0	82.4	17.5	15.3	9.6	6
Income below poverty threshold								
1990	17	10.0	8.7	7.8	38.8	17.9	11.4	9.6
2000	17.6	9.9	8.5	7.9	42.5	22.4	14.5	11.6
2010	22.4	13.5	11.9	10.8	43.7	23.2	15.4	12.4
2019	16.9	8.9	8.4	7.9	48.7	26.8	18.1	13.6

^5^,

**Table 6:** Sample characteristics and share in non-family households by age group, covariate, and census or survey year, females

	% of total sample				% living in non-family household			
	20–24	25–29	30–34	35–39	20–24	25–29	30–34	35–39
*University complete*								
1990	12.2	22.5	22.8	24.9	21.6	11.1	4.9	3
2000	13.7	29.9	29.2	26.3	22	10.9	4.4	2.6
2010	16.3	35	35.7	34.5	22.7	12.5	4.4	2.2
2019	21.1	39.9	42.9	42.4	21.9	13.9	5.4	2.8
*Attending school*								
1990	30.7	13.6	10.3	9.2	8.4	5.7	3.3	2.3
2000	34.6	15.1	9.3	7.1	9.4	6.3	3.4	2.7
2010	44.2	19.5	11.7	8.4	11	7.9	4	2.8
2019	42.2	16.1	9	6.5	12.6	9.5	4.9	3.6
*Employed*								
1990	66.5	69.9	69.4	72.2	13.2	7.5	4.1	2.7
2000	65.8	69.7	68.5	69.9	15.1	7.8	4.1	2.8
2010	62.3	69.7	69.4	69.8	16.7	9.5	4.4	2.8
2019	70.8	77.3	75.8	75.3	16.8	11.0	5.3	3.4
*Foreign-born*								
1990	10.5	11.4	11.3	11.2	12.2	7.9	5	3.7
2000	15.1	18.5	18.4	16.6	12.4	8.2	4.5	3.4
2010	13.3	18.7	22.7	24.1	14.2	8.9	5.1	3.5
2019	11.6	15.3	20.2	23.1	16.7	11.3	4.9	3.4
*Non-Hispanic white*								
1990	71.2	72.8	74.3	75.6	13.7	6.5	3.3	2.3
2000	61.6	62.4	65.1	68.3	16.7	7.4	3.5	2.6
2010	57.5	58.1	57.2	58.2	19.2	9.2	4.1	2.6
2019	53.1	54	55.3	55.6	20	11.1	5.2	3.3
*Single*								
1990	62.5	31.2	17.5	11.2	18	16	12.4	10.6
2000	68.8	38.2	21.7	14.9	19.2	15.1	11.3	9.8
2010	81.7	51.8	30.4	20.3	18.5	14.3	10	7.6
2019	86.4	61.5	37.8	25.5	18.4	15.1	10.5	8
*Living in metropolitan area*								
1990	80.6	79.3	76.9	75.3	14	7.3	4	2.8
2000	78.2	80.1	79.8	78.4	14.7	7.8	4	3
2010	79.8	81.1	80.4	79.8	16.2	9.3	4.6	2.9
2019	82.3	83.6	83.6	82.6	16.8	11.0	5.3	3.5
*Income below poverty threshold*								
1990	21	14.2	12.7	10.4	29.6	9.7	6.2	5.7
2000	24	14.6	12.6	11.3	30.6	11.2	7.15	6.7
2010	29.1	19.7	17.4	15.4	33.9	12.1	7.4	6.4
2019	21.9	14.6	13.5	12.9	41.2	16.8	9.5	8

^6^,

**Table 7:** Results of logistic regression of likelihood to live in a non-family household for males and females aged 20–24

	Odds Ratio (SE)			
	Males		Females	
Variable	Model 1	Model 2	Model 1	Model 2
*Year*				
1990 (reference)	1	1	1	1
2000	1.268 (0.002)	1.325 (0.002)	1.183 (0.002)	1.014 (0.002)
2010	1.277 (0.002)	1.072 (0.002)	1.350 (0.002)	0.890 (0.001)
2019	1.160 (0.001)	1.060 (0.002)	1.418 (0.002)	1.030 (0.002)
*Educational attainment*				
Primary or less (reference)		1		1
Secondary		1.416 (0.002)		1.792 (0.004)
University		2.742 (0.006)		3.619 (0.010)
School attendance		1.429 (0.002)		1.585 (0.002)
*Employment status*				
Employed (reference)		1		1
Unemployed		0.379 (0.001)		0.456 (0.001)
Inactive		0.553 (0.001)		0.566 (0.001)
Foreign-born		1.479 (0.002)		1.162 (0.002)
*Race and ethnicity*				
White (reference)		1		1
Non-Hispanic Black		0.344 (0.001)		0.266 (0)
Non-Hispanic Asian		0.660 (0.002)		0.864 (0.002)
Non-Hispanic other		0.577 (0.002)		0.602 (0.002)
Hispanic any race		0.516 (0.001)		0.427(0.001)
*Marital status*				
Single (reference)		1		1
Married		0.101 (0)		0.085 (0)
Divorced, separated, widowed		1.044 (0.004)		0.473 (0.001)
Residence in metropolitan area		1.548 (0.002)		1.665 (0.002)
*Poverty status*				
Below threshold (reference)		1		1
Income 100–200% of threshold		0.327 (0)		0.341 (0)
Income > 200% of threshold		0.075 (0)		0.075 (0)

*p* < 0.001 for all variables

7,

**Table 8:** Results of logistic regression of likelihood to live in a non-family household for males and females aged 30–34

	Odds ratio (SE)			
	Males		Females	
Variable	Model 1	Model 2	Model 1	Model 2
Year				
1990 (reference)	1	1	1	1
2000	1.262 (0.002)	1.124 (0.002)	1.057 (0.002)	0.926 (0.002)
2010	1.449 (0.003)	1.024 (0.002)	1.219 (0.003)	0.815 (0.002)
2019	1.656 (0.003)	1.078 (0.002)	1.479(0.003)	0.884 (0.002)
*Educational attainment*				
Primary or less (reference)		1		1
Secondary		0.983 (0.002)		1.033 (0.003)
University		1.289 (0.003)		1.508 (0.005)
School attendance		1.157 (0.003)		1.151 (0.003)
*Employment status*				
Employed (reference)		1		1
Unemployed		0.626 (0.002)		0.876 (0.003)
Inactive		0.611 (0.001)		0.730 (0.002)
Foreign-born		1.757 (0.003)		1.579 (0.004)
*Race and ethnicity*				
White (reference)		1		1
Non-Hispanic Black		0.659 (0.001)		0.389 (0.001)
Non-Hispanic Asian		0.945 (0.002)		1.071 (0.004)
Non-Hispanic other		0.933 (0.003)		0.838 (0.004)
Hispanic any race		0.970 (0.002)		0.707 (0.002)
*Marital status*				
Single (reference)		1		1
Married		0.059 (0)		0.036 (0)
Divorced, separated, widowed		0.852 (0.001)		0.565 (0.001)
Residence in metropolitan area		1.680 (0.003)		1.529 (0.004)
*Poverty status*				
Below threshold (reference)		1		1
Income 100–200% of threshold		0.603 (0.001)		0.700 (0.002)
Income > 200% of threshold		0.349 (0.001)		0.506 (0.001)

*p* < 0.001 for all variables

8,

**Table 9:** Results of logistic regression of likelihood to live in a non-family household for males and females aged 35–39

	Odds ratio (SE)			
	Males		Females	
Variable	Model 1	Model 2	Model 1	Model 2
*Year*				
1990 (reference)	1	1	1	1
2000	1.198 (0.003)	1.021 (0.002)	1.123 (0.003)	1.001 (0.003)
2010	1.408 (0.003)	0.957 (0.002)	1.134 (0.003)	0.811 (0.002)
2019	1.440 (0.003)	0.956 (0.002)	1.400 (0.004)	0.948 (0.003)
*Educational attainment*				
Primary or less (reference)		1		1
Secondary		0.888 (0.002)		0.956 (0.003)
University		0.878 (0.002)		1.100 (0.004)
School attendance		1.248 (0.004)		1.029 (0.003)
*Employment status*				
Employed (reference)		1		1
Unemployed		0.742 (0.002)		0.859 (0.004)
Inactive		0.667 (0.002)		0.835 (0.002)
Foreign-born		1.885 (0.004)		1.518 (0.005)
*Race and ethnicity*				
White (reference)		1		1
Non-Hispanic Black		0.802 (0.002)		0.426 (0.001)
Non-Hispanic Asian		0.905 (0.004)		1.176 (0.005)
Non-Hispanic other		0.880 (0.004)		0.790 (0.005)
Hispanic any race		1.103 (0.003)		0.707 (0.002)
*Marital status*				
Single (reference)		1		1
Married		0.058 (0)		0.037 (0)
Divorced, separated, widowed		0.874 (0.002)		0.613 (0.001)
Residence in metropolitan area		1.655 (0.004)		1.275 (0.003)
*Poverty status*				
Below threshold (reference)		1		1
Income 100–200% of threshold		0.586 (0.001)		0.612 (0.002)
Income > 200% of threshold		0.387 (0.001)		0.488 (0.001)

*p* < 0.001 for all variables

9
